# Prospect of Tellurium in High-Temperature Carburizing Gear Steels: An Industrial Study

**DOI:** 10.3390/ma18092162

**Published:** 2025-05-07

**Authors:** Jin Wang, Yun Bai, Wei Liu, Huiyu Xu, Qingsong Zhang, Guangwei Wang, Shufeng Yang, Jingshe Li

**Affiliations:** 1 School of Metallurgical and Ecological Engineering, University of Science and Technology Beijing, Beijing 100083, China; anhwangjin@163.com (J.W.); d202110112@xs.ustb.edu.cn (Y.B.); xuhuiyu@xs.ustb.edu.cn (H.X.); guangweiwang@ustb.edu.cn (G.W.); lijingshe@ustb.edu.cn (J.L.); 2 Jiangyin Xingcheng Special Steel Works, Co., Ltd., Wuxi 214400, China; zhangqingsong@citicsteel.com; 3 State Key Laboratory of Advanced Metallurgy, University of Science and Technology Beijing, Beijing 100083, China

**Keywords:** gear steel, high temperature carburizing, tellurium, inclusions, free-cutting

## Abstract

This work is a continuation of our previous research. We successfully produce low-carbon gear steel containing trace tellurium (Te) through industrial production line (EAF-LF-VD-CC), and we investigate the effects of a trace Te addition on the precipitation of MnS inclusions in sulfur-containing gear steel billets, the machinability of rods, and the high-temperature vacuum carburizing performance of rods. This study demonstrates that the addition of trace Te in steel can be achieved in industrial production without causing disruptions in the steelmaking process. The Te addition effectively induces spheroidization and refinement of MnS inclusions in industrial cast billets, showing good consistency with laboratory Te alloying experimental results. Furthermore, the Te addition reduces the deformation rate of MnS inclusions during industrial rolling processes. Benefiting from the spheroidization of MnS inclusions, the chip-breaking performance during the machining of Te-containing rods is significantly optimized, along with substantial improvement in machined surface roughness. The industrial rods exhibit excellent grain stability during 960 °C high-temperature vacuum carburizing, with carburizing rates significantly enhanced compared to conventional gear steels. This work comprehensively demonstrates the multifaceted effects of Te treatment on gear steel properties, particularly providing valuable references for developing high-temperature carburizing gear steels.

## 1. Introduction

Gears, as core fundamental components in mechanical transmission systems, are critical elements in industrial base materials [1]. Harsh service environments and the low-carbon development of industries are driving steel manufacturers to produce gear steels with ultra-high purity, excellent machinability, and suitability for high-temperature carburizing. Sulfur is generally removed in most steels due to its detrimental effects on plasticity and toughness [2]. However, for gear steels requiring precision machining, appropriately increasing sulfur content can enhance machinability [3]. This is attributed to sulfur forming MnS inclusions with manganese during steel solidification, where MnS plays a key role in improving machining performance. Nevertheless, MnS is soft and prone to deformation during rolling, thereby degrading mechanical properties. Higher sulfur content makes sulfide inclusions harder to control, resulting in more numerous, larger-sized, and higher-aspect-ratio sulfides in cast billets. These elongated sulfide inclusions undergo greater deformation during rolling, exacerbating material anisotropy [4,5,6].

Consequently, mitigating the detrimental effects of sulfide inclusions in sulfur-containing gear steels has become a focal point for metallurgical researchers in recent years. Studies indicate that the chemical modification of sulfide inclusions is a viable approach. Common modifiers include rare earth elements (RE) [7,8,9], magnesium (Mg) [10,11], and Te [12,13,14,15,16]. Zhuo et al. [17] demonstrated that adding varying Ce contents to rail steel alters the morphology of elongated MnS and irregular Al-Si-Ca-O inclusions. With increasing Ce content, the evolution sequence progresses as: MnS + Al-Si-Ca-O → Ce_2_O_2_S + MnS → Ce_2_O_2_S + MnS + Ce_2_S_3_ → Ce_2_O_2_S + Ce_3_S_4_ + Ce_2_S_3_ → Ce_2_O_2_S + Ce_3_S_4_ + CeS. In pipeline steels, Ce also influences the evolution sequence of sulfide and oxide inclusions [18]. Overall, rare earth elements are excellent modifiers for non-metallic inclusions, but industrial application challenges, such as nozzle clogging and RE burning [19], limit their widespread use. In previous research works [16,20], the surfactant Te has demonstrated a significantly positive influence on sulfide inclusions in steel. The mechanism of Te’s effect on sulfide inclusions has been partially elucidated in the studies [21,22], which collectively suggest that the formation of MnS-MnTe composite inclusions constitutes the primary reason for the enhanced spheroidization degree of inclusions in experimental steels. Shen et al. [22] systematically investigated the formation mechanisms of MnS-MnTe composite inclusions with distinct morphological characteristics under varying Te content conditions. At lower Te concentrations, the predominant structure manifests as core-shell composite inclusions comprising MnS cores enveloped by MnTe outer layers. Conversely, under conditions of higher Te content, the characteristic morphology transitions to MnS particles dispersed within the MnTe matrix. It should be noted, however, that these findings were obtained through laboratory-scale investigations, and practical industrial applications of Te remain conspicuously scarce.

The control of grain stability during carburizing heat treatment in gear steels has become a critical focus in composition design research. Alloying elements, such as Nb [23,24,25], Ti [26,27], B [28], and Mo [26], are commonly employed, with research strategies centered on introducing these elements to form second-phase particles. These particles provide significant pinning resistance to grain boundaries during carburizing heat treatment, thereby enhancing high-temperature grain stability. In our previous study [20], Te was found to markedly improve the thermal stability of steel grains, specifically by effectively suppressing austenite grain growth during isothermal heat treatment. This discovery offers strong support for advancing high-temperature carburizing processes. However, earlier work was conducted under laboratory conditions and lacked practical carburizing experiments.

Building upon prior research, this work implemented industrial-scale production trials of Te-treated gear steel. Purity assessments of cast billets and rods obtained from industrial trials, combined with high-temperature vacuum carburizing experiments, comprehensively evaluated the application potential of Te treatment in high-quality steel production. These findings provide valuable insights for developing sulfur-containing, easily machinable gear steels suitable for high-temperature carburizing.

## 2. Materials and Methods

### 2.1. Industrial Production

**Figure 1:**
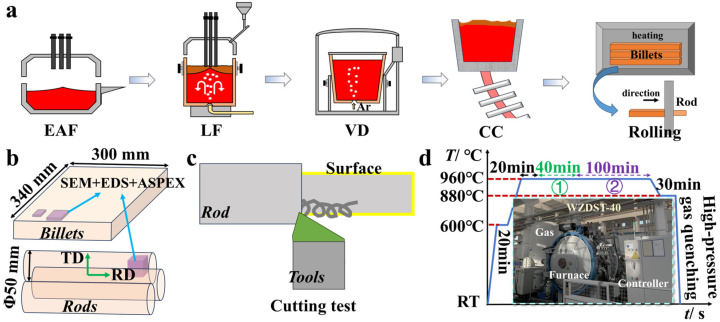
Experimental methods: (**a**) industrial production process; (**b**) industrial billet and rod sizes and sampling program; (**c**) cutting experiments; (**d**) high-temperature vacuum carburizing experiments.

The production processes of the two experimental steels are illustrated in Figure 1a. First, a specific ratio of molten iron and preheated scrap steel was charged into the electric arc furnace (EAF). After melting, the molten steel was tapped into a ladle. The ladle was then transferred to the ladle furnace (LF) station for refining and composition adjustment. Following the vacuum degassing (VD) process, the steel composition approached the target specifications, with most deoxidation products and impurities removed. Te alloying was performed after breaking vacuum in the VD process. Pure Te-containing cored wire was injected into the molten steel via a wire feeder. The entire process was completed without causing production fluctuations or significant fume emissions. The dimensions of the cast billets and rods are shown in Figure 1b. Samples from both heats (billets and rods) were collected for subsequent experiments and compositional analysis. The alloying elements in the experimental steels were determined using optical emission spectrometry. The oxygen (O) and nitrogen (N) contents were measured by an oxygen/nitrogen analyzer (TCH600, LECO Co., Ltd., St. Joseph, MI, USA), while carbon (C) and sulfur (S) concentrations were analyzed using a C/S analyzer. The chemical compositions of both experimental steels are presented in Table 1. The Te-treated steel contains 0.0051 wt% Te, and for ease of subsequent discussion, the Te-added steel is designated as “50Te”, whereas the Te-free steel is labeled as “Te-free”.

### 2.2. Inclusion Characterization and Rod Cutting Test

As shown in Figure 1b, metallographic samples (10 mm × 10 mm × 10 mm) and cylindrical rods (Φ16 mm × 200 mm) were sectioned from the experimental steel billets and rods for inclusion analysis and machining experiments. The morphological characteristics of inclusions were characterized using a field emission scanning electron microscope (FE-SEM, ZEISS Gemini SEM 300, Carl Zeiss AG, Oberkochen, Germany) and an optical microscope (Leica DML8A, Leica Microsystems GmbH, Wetzlar, Germany). Automated SEM was employed to comprehensively collect and statistically analyze the composition and morphology of all inclusions within a defined sample area. As illustrated in Figure 1c, machining tests were conducted on a DX6060 CNC engraving and milling machine (manufactured by Sinhong, Taizhou, China). The machining parameters included a spindle speed of 1500 r/min, a feed rate of 0.1 mm/min, and a total cutting depth of 1 mm. Surface roughness of the machined specimens was measured using an surface roughness tester (SJ-410, manufactured by Mitutoyo Corporation, Kawasaki, Kanagawa, Japan). Five random measurements were taken on each machined surface, and the average value was calculated.

### 2.3. High-Temperature Vacuum Carburizing Experiment

As shown in Figure 1d, high-temperature vacuum gas carburizing was conducted in a single-chamber vacuum furnace (WZDST-40, manufactured by China Academy of Machinery Beijing Research Institute of Mechanical & Electrical Technology Co., Ltd., Beijing, China). The furnace was equipped with two gas control systems, one for carburizing gas and another for quenching gas. The carburizing furnace was connected to an external gas control cabinet, allowing the precise adjustment of gas flow rates and ratios via high-precision flowmeters to meet process requirements. Specimens used for carburizing were cylindrical rods with a diameter of 12 mm and a length of 80 mm. The temperature control profile for the entire carburizing process is illustrated in Figure 1d. Stage ① represents the strong carburizing phase at 960 °C for 42 min. The carburizing gas medium was acetylene, with a pressure of 3 kPa and a flow rate of 8 L/min. Stage ② corresponds to the diffusion phase at 960 °C for 100 min. Subsequently, the temperature was reduced to 880 °C and held for 30 min, followed by high-pressure gas quenching using nitrogen at 6 bar. During actual quenching, the temperature dropped from 880 °C to 100 °C within approximately 7 min. To obtain accurate carbon concentration gradients, the rods underwent layer-by-layer turning (0.1 mm per layer) using a lathe, with machined chips collected for carbon content analysis via a carbon-sulfur analyzer. For data reliability, carbon concentration profiles were derived from two independently sampled rods, with each layer tested 2–3 times and averaged.

## 3. Results and Discussion

### 3.1. Effect of Te on MnS Inclusions Precipitation and Rolling Deformation in Industrial Cast Billets and Rods

**Figure 2:**
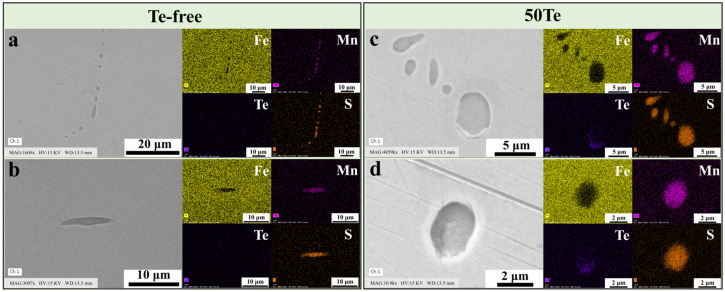
SEM maps and EDS results of sulfide inclusions in industrial cast billet: (**a**,**c**) aggregate distribution of inclusions; (**b**,**d**) individual inclusions.

**Figure 3:**
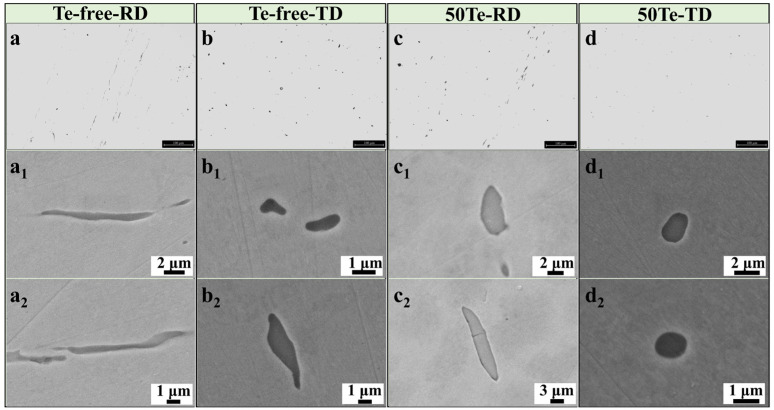
Morphology of MnS inclusions in industrial rods: (**a**–**d**) OM images; (**a_1_**–**d_1_**) SEM images; (**a_2_**–**d_2_**) SEM images.

Figure 2 illustrates the typical morphologies of MnS inclusions at the edge region of the cast billets. As shown in Figure 2a, MnS inclusions in the Te-free billet exhibit pronounced intergranular aggregation precipitation, consistent with findings reported in other studies [29]. This intergranular precipitation behavior is significantly influenced by sulfur content. However, since the sulfur content in both billets of this work shows no substantial difference, similar sulfide inclusion aggregation is also observed in the 50Te slab (Figure 2c). The rapid liquid-to-solid phase transformation of the cast billets under high cooling rates promotes the formation of MnS inclusions with a smaller equivalent circular diameter (ECD) and a lower two-dimensional aspect ratio (2D aspect ratio), particularly at the billet edges. Although the processing parameters of both steels are nearly identical, a comparative analysis of Figure 2b,d reveals that inclusions in the Te-free slab exhibit a larger ECD and a higher 2D aspect ratio compared to those in the 50Te billet. This indicates that the Te addition affects sulfide morphology starting from the initial solidification stage, aligning with our previous laboratory findings [16]. After heating and homogenization, the billets were subjected to rolling. As shown in Figure 1b, square billets with cross-sectional dimensions of 340 mm × 300 mm were rolled into round rods with a diameter of 50 mm. Figure 3 presents the composition and distribution characteristics of inclusions in the rods of both experimental steels. From Figure 3a–d, distinct differences in inclusion distribution across rod cross-sections are evident. In the rolling direction (RD), inclusions displayed pronounced orientation alignment, whereas their distribution appeared more random in the transverse direction (TD). This behavior arises from the soft nature of MnS inclusions, leading to significant deformation during hot rolling. As shown in Figure 3, inclusions in the Te-free rods exhibited an elongated morphology, while those in the 50Te rods experienced less deformation.

**Figure 4:**
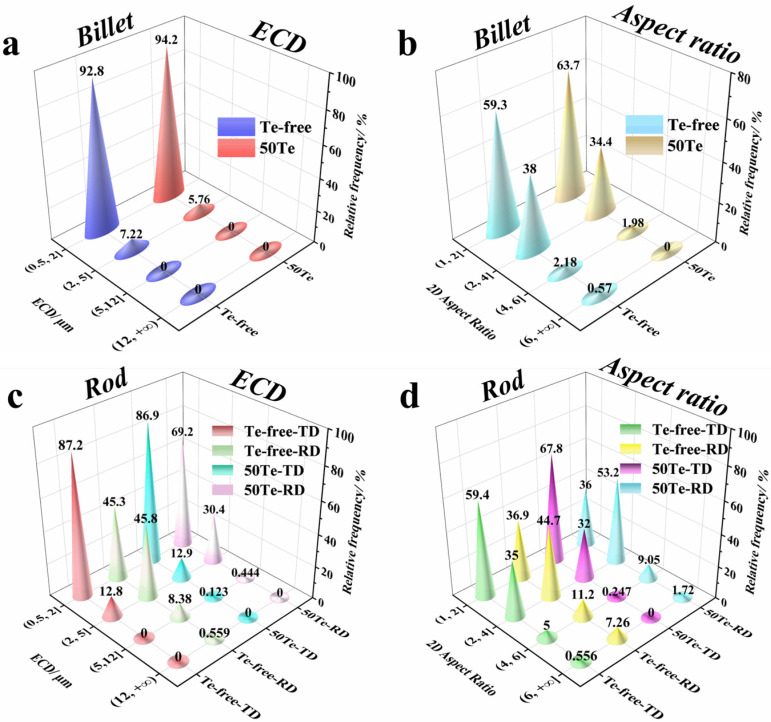
Quantitative results of the morphological characterization of inclusions in test steels: (**a**) ECD distribution of cast billets; (**b**) 2D aspect ratio distribution of cast billets; (**c**) ECD distribution of rods; (**d**) 2D aspect ratio distribution of rods.

To quantitatively compare MnS inclusion characteristics in the billets and rods of both experimental steels, automated scanning electron microscopy (SEM) was employed to scan and collect inclusion data. The statistical results are presented in Figure 4. Figure 4a,b shows the statistical information of MnS inclusions in the billets before and after Te treatment. The 2D aspect ratios of MnS inclusions in both billets were ≤5, indicating minimal presence of elongated inclusions. In detail, the 50Te billets exhibited a higher proportion of MnS inclusions within smaller 2D aspect ratio ranges, while the Te-free billets contained slightly more large-sized inclusions. Figure 4c,d summarizes inclusion characteristics across different rod cross-sections. For each steel, MnS inclusions in the rolling direction (RD) of the rods tended to exhibit larger ECD and higher 2D aspect ratios. Comparing the two steels, only 10.77% of MnS inclusions in the 50Te-RD samples had 2D aspect ratios > 4, whereas the Te-free-RD samples showed 18.46% in this range. Additionally, elongated inclusions exceeding 12 μm in ECD were observed in the Te-free-RD rods. These statistical results align with the analysis of Figure 3, demonstrating that the 50Te steel exhibits superior distributions in both 2D aspect ratios and the ECD of inclusions.

**Figure 5:**
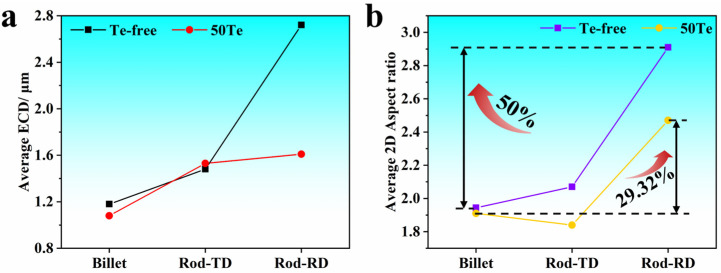
(**a**) Average ECD and (**b**) average 2D aspect ratio in industrial billets and rods.

To further analyze the MnS inclusion characteristics in the experimental steels from Figure 4, the 2D aspect ratio and ECD were averaged, with results summarized in Figure 5. As shown in Figure 5a, the 50Te billets exhibited a marginally smaller average ECD of inclusions compared to the Te-free billets. This difference arises from Te enrichment at the solidification front, which reduces surface tension during sulfide precipitation. The lowered surface tension enhances nucleation rates [16,30], resulting in a higher population of finer, near-spherical sulfide inclusions.

Consequently, Figure 5b reveals that the average 2D aspect ratio of inclusions in the 50Te billets was also slightly lower than in the Te-free counterparts. As demonstrated in prior studies, inclusions undergo substantial deformation during billet rolling. Figure 5 highlights that the disparity in MnS characteristics between the two steels predominantly occurs in the rolling direction (RD) of the rods. Specifically, Figure 5b shows that rolling increased the average 2D aspect ratio of inclusions in the Te-free steel from 1.94 to 2.91 (50% deformation rate), while the 50Te steel exhibited a significantly lower deformation rate of 29.32%. These findings confirm that the 50Te steel experienced markedly less inclusion deformation, further supported by the ECD variations depicted in Figure 5a. The enhanced rolling deformation resistance of sulfides in Te-treated steel may be attributed to two mechanisms. First, Te treatment promotes the formation of a Te-rich layer encapsulating MnS inclusions. During hot working, this layer generates a liquid phase that establishes a dynamic buffer zone in stress concentration regions. This buffer dissipates external load energy during rolling, effectively reducing stress transmission to the sulfide matrix and suppressing MnS deformation [14]. Second, the Te addition increases the intrinsic hardness of MnS inclusions. Wang et al. [31] employed nanoindentation to characterize the microhardness of MnS and Mn(S,Te) inclusions. Their results revealed a pronounced rightward shift in the load–displacement curve of Mn(S,Te) compared to MnS, indicating superior deformation resistance in Te-containing inclusions, which minimizes deformation during rolling.

### 3.2. Effect of Te on the Cutting Performance of Industrial Rods

**Figure 6:**
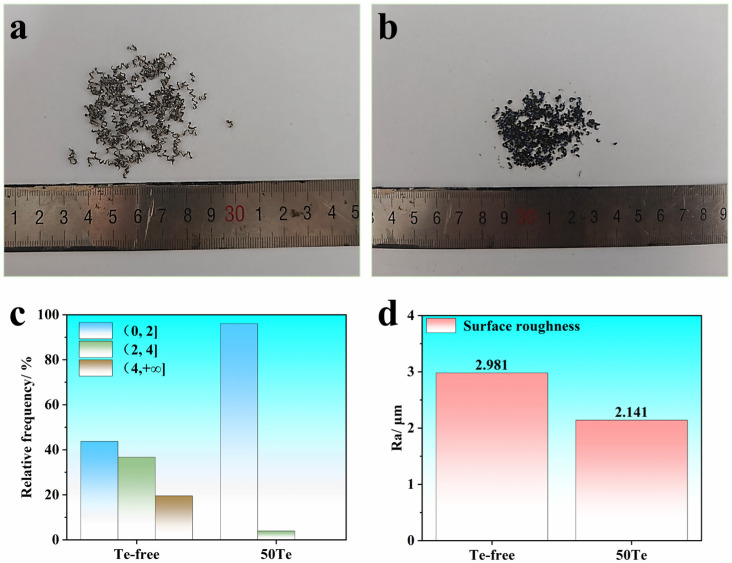
Results of rod cutting experiments: (**a**) test steel Te-free; (**b**) test steel 50Te; (**c**) mass percentage of different shapes of chips; (**d**) roughness of the cut surface.

Chip-breaking behavior during machining and post-machining surface roughness constitute critical technical indicators for evaluating machinability. In machining processes, chip morphology directly reflects the workability of experimental steels. It is generally accepted that an increased proportion of short helical chips benefits machining operations, whereas excessively long helical chips may cause chip evacuation difficulties, potentially necessitating process interruption for chip removal. Figure 6a,b presents the chip morphologies obtained from machining both experimental steel rods. The 50Te rods predominantly generated short helical chips, while the Te-free rods exhibited a higher proportion of long helical chips. Figure 6c provides a quantitative statistical analysis of chip types based on helical turns, with mass proportions calculated through sieved chip classification. The results demonstrate superior chip-breaking performance in 50Te rods during machining. Furthermore, Figure 6d compares the average surface roughness of machined specimens. The 50Te rods achieved a surface roughness of 2.141 μm, significantly lower than the 2.981 μm value for Te-free rods, indicating an enhanced surface finish in Te-treated steel.

**Figure 7:**
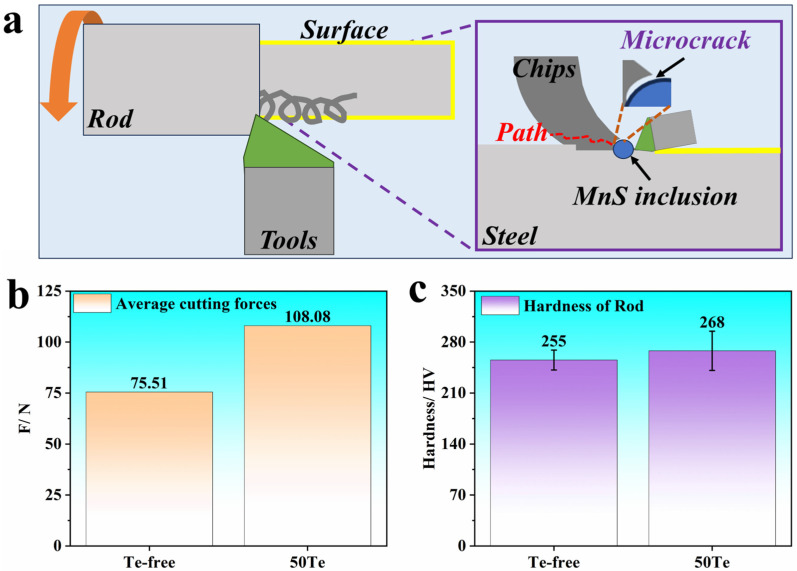
Mechanisms of optimization of cutting properties of industrial rods caused by the Te addition: (**a**) MnS inclusions inducing microcrack formation; (**b**) average cutting force during cutting; (**c**) hardness of industrial rods.

The mechanisms underlying the improved chip-breaking performance and surface finish induced by the Te addition can be elucidated through the characteristics of MnS inclusions. As demonstrated earlier, Te promotes the spheroidization of MnS inclusions in steel, and the machinability of sulfur-containing steels is predominantly governed by MnS inclusion behavior. As illustrated in Figure 7a, during machining, when cutting tools apply mechanical loads to the steel surface, pre-existing MnS inclusions alter local stress distribution. The elastic modulus mismatch between inclusions and the matrix induces stress concentration at inclusion–matrix interfaces under triaxial cutting forces. This stress concentration initiates microcracks that propagate along the maximum shear stress direction, ultimately leading to fracture. This process critically influences chip morphological evolution, transforming continuous multi-helical chips into single-helical or fragmented chips. Notably, spherical inclusions amplify stress concentration effects more significantly than elongated inclusions, thereby enhancing microcrack initiation and propagation during chip formation.

In addition to chip formation characteristics, Figure 7b presents the average cutting force during the machining process. From the cutting force perspective, the addition of Te increases the cutting force exerted on the tool, which represents an unfavorable parameter, as it may lead to reduced tool lifespan. To investigate the potential influence of excessive hardness on cutting force elevation, the hardness of both steel samples was measured using a VTD512 micro-Vickers hardness tester (manufactured by Beijing Woweitek Technology Co., Ltd., Beijing, China) under a test load of 300 gf with a dwell time of 15 s, as shown in Figure 7c. The 50Te steel exhibited marginally higher overall hardness compared to the Te-free steel, contributing partially to the observed cutting force increase. However, the limited hardness differential between the two steels necessitates further investigation into their microstructural characteristics in future work.

Furthermore, this experimental observation provides a basis for discussing the theoretical framework proposed in the studies [32,33]. Previous researchers hypothesized that surface-active agents could reduce shear strength, thereby decreasing cutting forces. This mechanism is attributed to the formation of ultra-thin adsorption films at matrix–heterophase interfaces (e.g., inclusions, carbides), which lower interfacial energy and consequently reduce shear strength. Contrary to these theoretical predictions, the average cutting forces measured in this work demonstrate an inverse trend, requiring more detailed exploration in subsequent research endeavors.

### 3.3. Effect of Te on High-Temperature Vacuum Carburizing Properties of Industrial Rods

**Figure 8:**
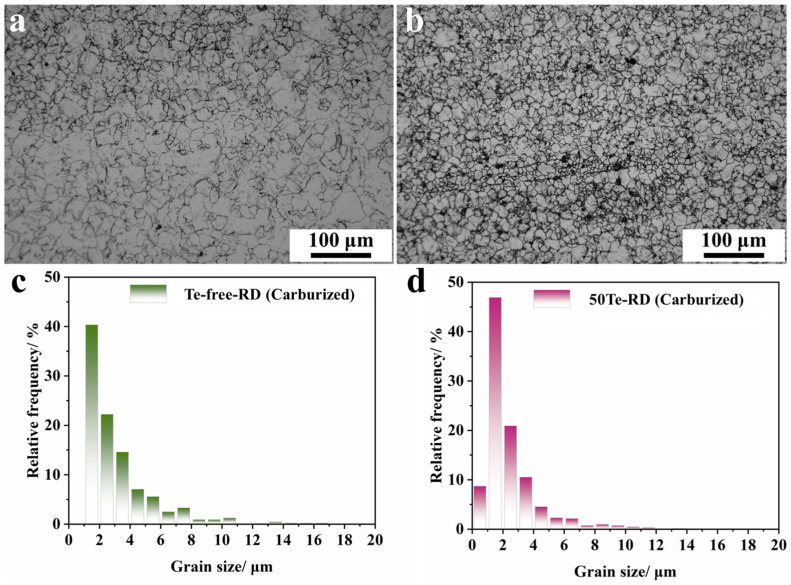
Proto-Austenitic grain characteristics of industrial rods after high-temperature (960 °C) vacuum carburizing: (**a**) OM map of the grains at 3 mm below the surface of Te-free industrial rods; (**b**) OM map of the grains at 3 mm below the surface of 50Te industrial rods; (**c**) grain size distribution of the RD direction of Te-free industrial rods obtained by EBSD; (**d**) grain size distribution of the RD direction of 50Te obtained by EBSD.

In our previous work, we demonstrated the grain refinement effect of Te during high-temperature pseudo carburizing of gear steel [20]. To elucidate the impact of Te-containing gear steel on efficient carburizing behavior in industrial production, high-temperature vacuum carburizing experiments were conducted in this study. Given the rapid grain coarsening typically observed during high-temperature carburizing of gear steels, the high-temperature grain stability of the 50Te steel was prioritized for investigation. Figure 8a,b presents optical micrographs (OM) of grain distribution at 3 mm below the surface layer for both steel variants. Microstructural analysis revealed that the 50Te steel demonstrates significantly refined and homogenized grain distribution after carburization, whereas the Te-free steel exhibits a more stochastic grain arrangement with localized heterogeneity. To quantitatively compare grain size distribution characteristics, electron backscatter diffraction (EBSD) was employed to statistically analyze grains along the rolling direction (RD) of carburized specimens, as shown in Figure 8c,d. The results revealed a significantly higher frequency of sub-2 μm grains in the 50Te steel compared to the Te-free counterpart. These findings collectively demonstrate that the Te-containing steel achieves superior grain size control and uniformity after identical high-temperature carburizing. This aligns with prior conclusions that a Te addition induces the precipitation of secondary phases, which effectively inhibit grain boundary migration during high-temperature carburizing heat treatment.

**Figure 9:**
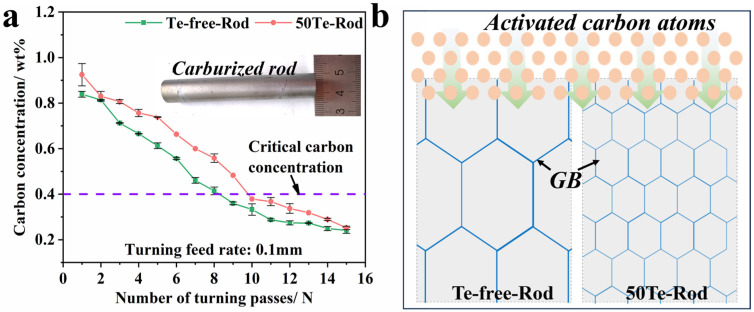
Carbon diffusion behavior of industrial rods and its influencing mechanism: (**a**) carbon concentration distribution pattern; (**b**) relationship between efficient carbon diffusion and grain refinement.

The primary objective of high-temperature carburizing is to enhance the carbon content in the workpiece surface layer, thereby improving surface properties, with the effective carburized case depth serving as a critical indicator of performance. Figure 9a illustrates the carbon concentration profiles obtained through layer-by-layer decarburization and the carbon content analysis of carburized steel rods. A carbon content threshold of 0.4 wt% is conventionally defined as the effective carburized layer criterion. Comparative analysis revealed that the 50Te steel exhibited a greater effective case depth than the Te-free steel, indicating superior carburizing efficiency under identical conditions. In addition, in comparison to the conventional gas carburizing experiments conducted by Miao et al. [34] on 20MnCrS5 gear steel (about 175 min of carburizing at 930 °C), the present study obtained a greater depth of carburized layer than that by vacuum carburizing for a shorter period of time (42 min of strong carburizing and 100 min of diffusion at 960 °C). This indicates that Te-containing gear steels can obtain better carbon concentration gradients under high-temperature vacuum low-pressure carburizing process, and this better effective depth of the carburized layer is the key to improve the service life of gears in complex service environments.

To precisely predict carbon content distribution at varying depths, we fitted the experimental data in Figure 9a. Using Origin 2018 software (developed by OriginLab Corporation, Northampton, MA, USA), mathematical relationships between carbon content (y) and depth (x) were fitted for both steels, as represented by Equations (1) and (2) in Figure 9a. Both equations achieved coefficient of determination (R^2^) values ≥ 0.99, confirming the high accuracy of the fitted correlations between carbon content and carburizing depth. From the above equation, the effective carburizing layer depths of the two test steels can be calculated to be 0.86 mm and 0.904 mm, respectively, and the results further confirm the efficient carbon diffusion behavior of the new gear steel under the same carburizing process.

y = −0.4635x^3^ + 1.2669x^2^ − 1.6737x + 0.8391, R^2^ = 0.993 (1)

y = −0.706x^3^ + 1.692x^2^ − 1.954x + 0.9247, R^2^ = 0.994 (2)

The enhanced carburization efficiency in Te-treated experimental steels can be explained through the analysis of carbon diffusion behavior. When maintaining identical carburizing parameters (thereby eliminating temperature effects on atomic diffusion coefficients), the material’s intrinsic microstructural characteristics become the determining factor for carburization kinetics. Grain boundaries are widely recognized as preferential diffusion pathways due to their structural defect nature, where carbon atoms exhibit higher mobility compared to lattice diffusion within grains. As demonstrated in Figure 9b, coarser grain structures contain relatively fewer grain boundaries, forcing carbon atoms to traverse longer intracrystalline diffusion paths. Conversely, refined grain structures provide an increased grain boundary density, enabling more efficient carbon transport through these enhanced diffusion channels. The microstructural evidence presented in Figure 8 confirms that the 50Te steel maintains finer grains than its Te-free counterpart after carburization. This grain refinement effect significantly augments the available diffusion pathways through grain boundary networks, thereby establishing superior carburization kinetics in the 50Te steel.

## 4. Conclusions

This work produced Te-containing 20MnCrS5 gear steel on a 100 t EAF-LF-VD-CC industrial production line. The metallurgical quality and high-temperature carburizing performance of the Te-treated gear steel were fully evaluated using a variety of experimental techniques. The main conclusions are as follows:

(1) Feeding Te-containing cored wire into the molten steel using a wire feeder is an effective way to add Te in industrial production. Choosing to feed the wire after the ladle refining process can achieve a good alloy recovery rate without affecting the on-site production.
(2) Te showed similar results in industrial production to those observed in laboratory-scale studies. Te can promote the precipitation of MnS inclusions in the billet with a smaller size and a lower aspect ratio. After hot rolling, the rolling deformation rate of MnS inclusions in the Te-containing gear steel rod was 29.32%, lower than that in the Te-free gear steel rod. The stress buffering provided by the MnTe phase during rolling and the increased hardness of the MnS-MnTe inclusions are the key factors of the reduced rolling deformation.
(3) The addition of Te can effectively optimize chip breaking behavior during the machining of the Te-containing industrial rod. The globular MnS inclusions act as stress concentration sources at the interface, which is the main reason for the large production of short spiral chips during the machining of Te-containing steel. In addition, the surface roughness of the Te-containing industrial rod after machining is lower, which is beneficial for improving the machining accuracy of gear components.
(4) The vacuum carburizing test at 960 °C verified the role of Te in stabilizing high-temperature grain growth in gear steel. The grain structure of the Te-containing gear steel rod after high-temperature carburizing is more refined and uniformly distributed compared to that of the Te-free gear steel rod. In terms of carburizing layer depth, the fine-grained structure of the Te-containing gear steel rod provides more grain boundary channels for high-temperature carbon diffusion, ultimately resulting in a more excellent carbon concentration gradient.

## Figures and Tables

**Table 1:** Chemical composition of steel.

Steel/Composition	C	Si	Mn	P	S	Cr	Al	O	N	Te
Te-free	0.201	0.134	1.2	0.019	0.02	1.19	0.029	<0.001	<0.015	-
50Te	0.197	0.112	1.0	0.015	0.02	1.13	0.024	<0.001	<0.015	0.0051

## Data Availability

The original contributions presented in this study are included in the article. Further inquiries can be directed to the corresponding author.

## References

[1] Zhang Z., Wu Z., Yuan Y., Wang X., Tian Y. (2024). Microstructure Evolution and Mechanical Properties of High-Temperature Carburized 18Cr2Ni4WA Steel. Materials.

[2] Tinoco H.A., Fintova S., Heikkila I., Herrero D., Vuoristo T., Dlouhy I., Hutar P. (2022). Experimental and numerical study of micromechanical damage induced by MnS-based inclusions. Mater. Sci. Eng. A.

[3] Akasawa T., Sakurai H., Nakamura M., Tanaka T., Takano K. (2003). Effects of free-cutting additives on the machinability of austenitic stainless steels. J. Mater. Process. Technol..

[4] Shao X.-j., Wang X.-h., Ji C.-x., Li H.-b., Cui Y., Zhu G.-s. (2015). Morphology, size and distribution of MnS inclusions in non-quenched and tempered steel during heat treatment. Int. J. Miner. Metall. Mater..

[5] Wu M., Fang W., Chen R.-M., Jiang B., Wang H.-B., Liu Y.-Z., Liang H.-L. (2019). Mechanical anisotropy and local ductility in transverse tensile deformation in hot rolled steels: The role of MnS inclusions. Mater. Sci. Eng. A.

[6] Temmel C., Ingesten N.-G., Karlsson B. (2006). Fatigue anisotropy in cross-rolled, hardened medium carbon steel resulting from MnS inclusions. Metall. Mater. Trans. A.

[7] Luo S., Shen Z., Yu Z., Wang W., Zhu M. (2021). Effect of Ce addition on inclusions and grain structure in gear steel 20CrNiMo. Steel Res. Int..

[8] Wang X., Wu Z., Li B., Chen W., Zhang J., Mao J. (2024). Inclusions modification by rare earth in steel and the resulting properties: A review. J. Rare Earths.

[9] Zhao L., Yang J., Fu X. (2024). Effect of Ce Content on Modification Behavior of Inclusions and Corrosion Resistance of 316L Stainless Steel. Materials.

[10] Su L., Tian J., Hu S., Lv M., Li X., Qu T., Wang D., Zhan T. (2023). Effect of Ca/Mg on distribution and morphology of MnS inclusions in 45MnVS non-quenched and tempered steel. Metals.

[11] Ji S., Zhang L., Wang X. (2022). Effect of magnesium on inclusions in a high sulfur steel. Metall. Mater. Trans. B.

[12] Gui L., Long M., Chen H., Chen D., Duan H., Huang Y., Liu T. (2018). Effect of MnS precipitation on solute equilibrium partition coefficients in high sulfur steel during solidification. J. Mater. Res..

[13] Huang Q., Ren Y., Luo Y., Ji S., Zhang L. (2022). Deformation of MnS–MnTe Inclusions in a Sulfur-Containing Free-Cutting Steel with Tellurium Treatment. Metall. Mater. Trans. B.

[14] Katoh T., Abeyama S., Kimura A., Nakamura S. (1982). A study on resulfurized free-machining steel containing a small amount of tellurium. Denki Seiko (Electr. Furn. Steel).

[15] Liu B., Li Y., Hu T., Xu X., Liu N., Fu J. (2023). The Forms and Evolution of Free-Cutting Phase in Bi–Te–S Free-Cutting Steel. Steel Res. Int..

[16] Wang J., Bai Y., Zhang F., Qi Z., Liu W., Liu Q., Yang S., Li J. (2024). Theoretical precipitation modeling and multidimensional characterization of sulfide inclusions in tellurium-containing steels. Mater. Charact..

[17] Zhuo C., Liu R., Zhao Z., Zhang Y., Hao X., Wu H., Sun Y. (2022). Effect of Rare Earth Cerium Content on Manganese Sulfide in U75V Heavy Rail Steel. Metals.

[18] Cheng W., Song B., Yang Z., Mao J. (2022). Effect of rare earth Ce on modifying inclusions in Al-killed X80 pipeline steel. Trans. Indian Inst. Met..

[19] Wang Y., Song G., He J., Zhang D., Fu J., Shen P. (2023). Influence Factors of SEN Clogging of Rare-Earth Steel Continuous Casting and Solve Ideas of Japanese Steel Enterprises. Steel Res. Int..

[20] Bai Y., Wang J., Liu W., Wang M., Zhang J., Yang S., Zhang Q., Li J. (2024). Tellurium-Induced Reduction in Heat Susceptibility of Gear Steel During High-Temperature Carburizing. Metall. Mater. Trans. B.

[21] Wang F., Guo H., Liu W., Yang S., Zhang S., Li J. (2019). Control of MnS Inclusions in High- and Low-Sulfur Steel by Tellurium Treatment. Materials.

[22] Shen P., Yang Q., Zhang D., Yang S., Fu J. (2018). The Effect of Tellurium on the Formation of MnTe-MnS Composite Inclusions in Non-Quenched and Tempered Steel. Metals.

[23] Yang Y.-h., Wang M.-q., Chen J.-c., Dong H. (2013). Microstructure and Mechanical Properties of Gear Steels After High Temperature Carburization. J. Iron Steel Res. Int..

[24] He G., Zhang N., Wan S., Zhao H., Jiang B., Liu Y., Wu C. (2022). The Carburizing Behavior of High-Temperature Short-Time Carburizing Gear Steel: Effect of Nb Microalloying. Steel Res. Int..

[25] Zhang G.-q., He X.-f., Zhang Q.-z., Wang W.-j., Wang M.-q. (2021). Comparison of microstructure and heat treatment distortion of gear steels with and without Nb addition. J. Iron Steel Res. Int..

[26] Tang E., Yuan Q., Zhang R., Zhang Z., Mo J., Liang W., Xu G. (2023). On the grain coarsening behavior of 20CrMnTi gear steel during pseudo carburizing: A comparison of Nb-Ti-Mo versus Ti-Mo microalloyed steel. Mater. Charact..

[27] An X., Tian Y., Wang H., Shen Y., Wang Z. (2019). Suppression of austenite grain coarsening by using Nb–Ti microalloying in high temperature carburizing of a gear steel. Adv. Eng. Mater..

[28] He G., Feng Y., Jiang B., Wu H., Wang Z., Zhao H., Liu Y. (2023). Corrosion and abrasion behavior of high-temperature carburized 20MnCr5 gear steel with Nb and B microalloying. J. Mater. Res. Technol..

[29] Zheng Q.w., Gao X.y., Zhang L.f. (2024). Effect of Sulfur Content on Precipitation Behavior of Dendrite Sulfide Inclusion in Continuous Casted 20CrMnTi Gear Steel. Steel Res. Int..

[30] Jeong J., Park D., Shim S., Na H., Bae G., Seo S.-J., Lee J. (2020). Prediction of Behavior of Alumina Inclusion in Front of Solid–Liquid Interface in SPFH590 Steel. Metall. Mater. Trans. B.

[31] Wang M., Bai Y., Liu W., Yang S., Sun Y., Wang J., Li J. (2025). Effect of tellurium treatment on wear resistance and inclusions deformation of 20MnCrS5 gear steel. Iron Steel.

[32] Yaguchi H., Onodera N. (1988). The effect of tellurium on the machinability of AISI 12L14+ Te steel. Trans. Iron Steel Inst. Jpn..

[33] Shen P., Yang Q.-k., Zhang D., Wu Y.-x., Fu J.-x. (2018). Application of tellurium in free-cutting steels. J. Iron Steel Res. Int..

[34] Shan M., Jiangang W., Dongying J., Huijun Z., Mengyuan Y. (2020). Improvement and analysis of fatigue strength for mild steel 20MnCrS5 during carburizing and quenching. Mater. Sci..

